# Sleep Deficiency and Breast Cancer Risk Among Postmenopausal Women in the California Teachers Study (CTS)

**DOI:** 10.1007/s10552-020-01349-2

**Published:** 2020-09-26

**Authors:** S Hurley, D Goldberg, J Von Behren, J Clague DeHart, S Wang, P Reynolds

**Affiliations:** 1 Department of Epidemiology and Biostatistics, University of California San Francisco, Berkeley, CA, USA; 2 School of Community and Global Health, Claremont Graduate University, Claremont, CA, USA; 3 Division of Health Analytics, Department of Computational and Quantitative Medicine, Beckman Research Institute, City of Hope Comprehensive Cancer Center, Duarte, CA, USA

**Keywords:** Sleep, Breast Cancer, Etiology, Case-Control

## Abstract

**Purpose:**

There is provocative, yet inconsistent, evidence that sleep deficiency may influence the development of breast cancer. The purpose of this study was to evaluate the risk of breast cancer associated with sleep deficiency among postmenopausal women in the California Teachers Study (CTS).

**Methods:**

We conducted a case-control study of 2,856 invasive breast cancer cases and 38,649 cancer-free controls, nested within the CTS. Self-administered questionnaires were used to ascertain several components of sleep deficiency, including quality, latency, duration, disturbance and use of sleep medications. Additionally, a Global Sleep Index (GSI) was created by summing the individual sleep components and categorizing into quartiles. Multivariable logistic regression analyses were used to estimate odds ratios and 95% confidence intervals (OR, 95% CI).

**Results:**

Increased breast cancer risks were associated with sleep deficiency. With the exception of duration, linear increases in risk were associated with all the other individual components of sleep deficiency (p-trend ≤0.002). The OR for the highest GSI quartile vs. lowest was 1.24, 95% CI: 1.12 – 1.38; p-trend <0.001).

**Conclusions:**

Sleep deficiency may be a risk factor for breast cancer. Additional prospective studies and those aimed at elucidating underlying mechanism are warranted.

## INTRODUCTION

The CDC has declared insufficient sleep to be a “public health epidemic,” noting that an estimated 50–70 million US adults have sleep or wakefulness disorders.[1, 2] Inadequate sleep has been linked to a number of chronic conditions including cardiovascular disease, obesity and diabetes, as well as to overall increases in mortality.[2, 3] Bolstered by the recognition of circadian disruption as a probable human carcinogen by the International Agency for Research on Cancer (IARC) in 2010,[4, 5] the role of sleep in the development of cancer has garnered increasing attention over the last decade.[2, 6] Beyond its integral role in helping to maintain circadian rhythms, sleep has been shown in laboratory studies to play a fundamental role in regulating key processes critical to carcinogenesis, including cellular replication and proliferation, inflammation, and immune surveillance.[7, 8] Sleep may be particularly germane to breast cancer risk as insufficient sleep has been shown to influence estrogen signaling pathways by melatonin suppression.[9–12] The degree to which such effects may translate to breast cancer risk in humans remains unclear.[15–20]

There are many dimensions of sleep and the field of sleep research is filled with competing terminology. The predominant construct, however is that of ‘sleep deficiency,’ defined as a “deficit in the quantity or quality of sleep obtained versus the amount needed for optimal health, performance, and well-being.”[21] Sleep deficiencies can have multiple components, including: insufficient duration of sleep (sleep deprivation); poor sleep efficiency characterized by long latency (i.e., taking a long time to fall asleep) and disturbance (i.e., waking frequently and not falling back to sleep quickly/easily); inappropriate timing of sleep (out of sync with the body’s natural clock or circadian rhythm); and sleep disorders, such as apnea, that interfere with adequate duration and/or sleep quality.

Epidemiologic evidence for an etiologic link between sleep deficiency and cancer is sparse and inconsistent.[15, 16, 18] While a number of studies have provided provocative evidence for a link between sleep apnea and risk for a variety of cancers (including breast cancer), results have not been consistent, and have varied by cancer site.[22–26] Studies focused on breast cancer predominantly have evaluated sleep duration and have yielded inconsistent findings with some reporting elevated risks associated with short sleep duration,[27–29] some with long sleep duration,[30–32] and some reporting no effect.[33–35]. Data from the few breast cancer studies that have evaluated other components of sleep have provided some suggestive, though very limited, evidence that other dimensions of sleep deficiency may play a role in breast cancer development.[16, 36]

The objective of this study was to evaluate the risk of breast cancer associated with several dimensions of sleep deficiency in a population of postmenopausal California women.

## METHODS

### Study Population

This is a case-control study nested within the California Teachers Study (CTS). The CTS is a prospective cohort study of female California professional school employees, specifically designed to study breast cancer risk [37]. In 1995 and 1996 over 133,000 women aged 22 to 104 were enrolled in the CTS by responding to a survey that was mailed to all active and retired female members of the California State Teachers Retirement System. Upon entry into the study, all participants provided informed consent to use their data for research purposes such as this study. Subsequent to enrollment, two CTS participants requested to be withdrawn from the study and are not included in the present analysis. The CTS has been actively followed for cancer diagnosis, death and change of address from its inception, as described previously [37]. Upon entry into the cohort, CTS members completed a baseline survey that included questions on reproductive history, personal and family medical history, health behaviors and other lifestyle factors. Five subsequent mailed questionnaires were administered to update the baseline data and collect new information on exposures, risk factors, and health outcomes of emerging interest. Detailed questions about sleep were included on the fifth CTS Questionnaire (Q5), administered in 2012–2015. Overall, approximately 60% of CTS members who received the Q5 survey responded, 99% of whom answered at least one question regarding sleep. A full description of the CTS creation and its characteristics are described elsewhere [37].

### Ascertainment of Breast Cancer Cases and Controls

Cases and controls for the current analysis were drawn from 44,480 postmenopausal CTS participants who provided sleep information on the CTS Q5, were under the age of 90 at the time they responded to the CTS Q5, had no history of breast cancer prior to CTS enrollment, and had resided in California continuously from baseline through Q5. Incident cases of primary invasive breast cancer (SEER site = 26000) diagnosed from baseline through completion of the CTS Q5 were identified by annual linkages of the CTS to the California Cancer Registry (CCR). Case ascertainment for the CCR is estimated to be 99% complete [38]. Participants diagnosed with *in situ* cancer of the breast were excluded. Remaining CTS participants without a breast cancer diagnosis served as controls, excluding those with diagnoses of other invasive cancers (n=2,975). This resulted in the identification of 2,856 cases of primary invasive breast cancer and 38,649 cancer-free women who served as controls for the current analysis.

### Assessment of Sleep Characteristics

Sleep characteristics were derived from CTS Q5 responses (available online at: https://www.calteachersstudy.org/past-questionnaires). Questions were adapted from the Pittsburg Sleep Quality Index (PSQI), a standardized and validated self-administered questionnaire developed for the assessment of subjective sleep quality and widely-used in health studies [39–41]. The PSQI is comprised of 19 self-rated questions. The 19 questions are scored and combined to form seven components (quality, latency, duration, disturbance, efficiency, daytime dysfunction and medication use). The seven components are then summed to create the PSQI global score. Due to space constraints, we were not able to include all 19 of the PSQI questions on the CTS Q5. Specifically, we did not include questions that captured sleep efficiency or daytime dysfunction. To create a modified summary global sleep index (GSI) we adapted the PSQI approach, scoring each of the five components for which we had information (quality, latency, duration, disturbance, and medication use) from 0 to 3, with 0 being best and 3 being the worst. We then summed the individual scored components to generate a continuous variable with discrete whole number values ranging from zero to 15, which was then converted to an ordinal variable based on the quartile distribution, with the lowest quartile representing the best sleep. The GSI was not generated for approximately 3% of participants who were missing or had invalid responses for any of the five individual components.

To maintain consistency with the PSQI, the questions were asked about sleep during the prior month. Furthermore, participants were asked if the characteristics they reported for the prior month were typical of sleep during other periods during their life (including the prior year, 2–5 years prior, 6–10 years prior and 11 or more prior years).

### Assessment of Covariates

Information on breast cancer risk factors was ascertained by self-report on the CTS questionnaires. Factors included age, race, family history of breast cancer, parity, age at first full term pregnancy, age at menopause, age at menarche, lactation history, hormone therapy use, alcohol consumption, smoking, household income, and neighborhood socioeconomic status. We also considered information from the CTS questionnaires on factors potentially related to sleep deficiency including body mass index (BMI), physical activity, marital status, and current medication use (including depression medications, prescription pain medications, and non-steroid anti-inflammatory drugs (NSAIDs)). Comorbidities were defined as having reported ever receiving a physician diagnosis of diabetes, chronic obstructive pulmonary disease (COPD), Parkinson’s disease, depression, chronic fatigue syndrome (CFS), Lupus, inflammatory bowel disease (IBD), Crohn’s Disease, and multiple sclerosis. From these data, we created a comorbidity index by summing the number of comorbid conditions and then categorizing as none, one, two, or three or more. Information on chronotype was collected on the CTS Q5 using an abbreviated version of the Horne-Ostberg Morningness-Eveningness Questionnaire (MEQ), a standardized and validated survey instrument used to characterize a person’s underlying circadian rhythm [42–45]. These questions were used to characterize participants into five chronotypes: definite morning, more morning than evening, neither morning nor evening, more evening than morning, definite evening. Details of the assignment of chronotype are described in an earlier analysis of chronotype and breast cancer in this study population [46].

### Statistical Analysis

The risk of breast cancer associated with sleep deficiency was evaluated through unconditional logistic regression analyses. Regression models were run using PROC LOGISTIC in the SAS/STAT software version 9.4 of the SAS system (28) to estimate odds ratios (ORs) and 95% confidence intervals (CIs) separately for each of the individual components of sleep deficiency (quality, latency, disturbance, duration, and medication use) as well as for our global sleep index (GSI). A test for linear trend was performed with categories of each sleep variable modeled as an ordinal variable. Initial models were adjusted for only age and race. Fully-adjusted multivariable models were built via a two-step process. Starting with the full set of potential covariates, a backwards elimination approach was used, starting with a model that included all potential covariates and forced inclusion of the sleep variable, age and race. All factors for which the p-value for the Wald chi-square was < 0.10 were kept as covariates. We then further evaluated potential confounding by adding each of the excluded variables back into the model one at a time keeping those that changed the estimated odds ratio for the sleep variable by 10% or more. While we conducted this process separately for each sleep variable, it resulted in the same set of covariates for all sleep variables. The set of covariates included in our final multivariable models appear in the footnote of Table 4.

To evaluate potential effect modifiers, we conducted analyses stratified by BMI (< 25 kg/m^2^, 25–29 kg/m^2^, ≥ 30 kg/m^2^), age (40–64 years, 65–79 years, 80–89 years) and chronotype (morning type, evening type, neither type). Tests for statistical interactions were calculated based on the p-value of likelihood ratio tests comparing nested models with and without a multiplicative interaction term for each of these variables and the sleep variables. To evaluate whether risks differed between cases with hormonally responsive tumors (estrogen receptor positive (ER+) or progesterone receptor positive (PR+)) and non-responsive tumors (estrogen receptor negative (ER−) and progesterone negative tumors (PR−)) we conducted multivariable polytomous logistic regression.

Because information on sleep was ascertained post-diagnosis for cases, we conducted a number of sensitivity analyses to address the potential for reverse causality. We repeated our analyses, excluding the 178 and 363 breast cancer cases that were diagnosed within one and two years prior to completing Q5, respectively. We also repeated our analyses restricted to the subset of participants who indicated that their sleep habits had not change recently (i.e. that their reported sleep was typical of the past year) and restricted to those with long-term sleep stability (i.e. that their reported sleep was typical of at least the prior 5 years).

## RESULTS

The characteristics of the study population, overall and by case control status, are presented in Table 1. Participants ranged in age from 40 to 89 years of age, with the majority (68%) falling between the ages of 60 and 79. Similar to the full CTS cohort, the study population was predominantly non-Hispanic White (87%). Overall, the reproductive and behavioral characteristics of the study population generally mirror those of the full CTS cohort. Given the large sample size, the distribution of a number of factors statistically differed between cases and controls (p<0.05) but the magnitude of differences was generally quite small.

Reported sleep characteristics are summarized in Table 2. Overall the majority of participants reported very good (30%) or fairly good sleep (54%). Seven hours of sleep was the most common duration, reported by 42% of respondents. Consistent with national survey data [47], slightly more than a quarter reported insufficient sleep durations (i.e. < 7 hours). Nearly half (47%) reported falling asleep within 15 minutes of going to bed while 4% reported that it took more than an hour. About a quarter of respondents (22%) reported no sleep disturbance in the last month, while a similar proportion (20%) reported experiencing sleep disturbance three or more times a week over the last month. Nearly a third of participants (31%) reported taking some kind of sleep medication in the last month. Other than for sleep quality, the distribution of all individual components of sleep deficiency, as well as of the GSI, differed between cases and controls (p< 0.05), with cases reporting worse sleep than the controls (Table 2). A majority of women indicated that their reported sleep characteristics over the past month were typical of the past year (88%) or past two to five years (72%). The distribution of sleep stability did not differ between cases and controls, other than a marginally higher proportion of controls reporting that their recent sleep was indicative of their sleep 11 or more years ago (43% vs. 41%, p=0.02).

The correlation matrix for the sleep variables is presented in Table 3. Statistically significant positive correlations were observed between all sleep variables. Among the individual components of sleep deficiency, disturbance and quality were the most highly correlated (r=0.66). Sleep medication was the least correlated with the other individual sleep variables, with correlation coefficients all < 0.30. Correlations of the individual components with the GSI ranged from 0.55 for sleep medication to 0.79 for sleep disturbance.

The estimated risks of breast cancer associated with sleep deficiency are summarized in Table 4. Multivariable adjusted odds ratios (OR_adj_) were generally similar to those generated from the age- and race-adjusted models. Risks of breast cancer were significantly associated with the GSI such that women with the worst GSI (highest quartile) had an approximately 25% greater risk of breast cancer compared to women in the lowest GSI quartile (OR_adj_= 1.24, 95% CI: 1.12 – 1.38, p-trend< 0.001)). With the exception of sleep duration, increased risks of breast cancer were also observed for each of the individual components of sleep deficiency. Compared to those who reported very good sleep quality, respondents who reported fairly or very bad sleep quality had an approximate 20- to 30-percent increased risk of breast cancer (p-value for trend ≤ 0.002). Breast cancer risk also significantly increased with greater sleep latency and disturbance (p-trend < 0.001). Women who reported taking sleep medications in the last month had greater risks of breast cancer compared to women who did not take any (p-trend <0.001).

Stratified analyses revealed no significant differences by categories of BMI or age (*data not shown*). Analyses stratified by chronotype suggested some differential risks associated for sleep disturbance (p-value for interaction = 0.032) and sleep medications (p-value for interaction < 0.001) (Supplemental material, Table S1). Results from our polytomous regression analyses yielded generally similar risk estimates for hormonally responsive and non-responsive tumors; these analyses however were hindered by the small number of cases with ER-/PR-tumors (Supplemental material, Table S2).

On average, cases were diagnosed 8.2 years prior to reporting their sleep characteristics on the CTS Q5 (range = 1 day to 17.5 years). The median time interval from case diagnosis to CTS Q5 completion date did not significantly differ by reported sleep characteristics for any of the individual components of sleep (Wilcoxon Rank Sum test p-values >0.05). Although cases with the worst GSI (highest quartile) were significantly more likely to be recently diagnosed than women in the lowest quartile (Wilcoxon Rank Sum test p-value = 0.015), the magnitude of case control differences were quite modest (median time interval from diagnosis to Q5 was 8.0 vs 9.1 years, respectively). Results of our sensitivity analyses are summarized in Table 5. When we repeated our regression analyses excluding cases who were diagnosed shortly before (one year and two years before) reporting their sleep characteristics on the CTS Q5, the estimates of risk for the GSI were essentially the same as those reported in the full study population. When we restricted our sample to the 36,542 women who reported no recent changes in sleep (i.e. reported sleep was typical of the past year), the estimated risks were similar to those reported in the full study population. Likewise, when we restricted our analyses to the 28,714 women with long-term sleep stability (i.e. reported sleep was typical of at least the past five years), risk estimates for the GSI were similar, albeit slightly attenuated. Sensitivity analyses focused on the individual sleep deficiency components produced similar results (*data not shown*).

## DISCUSSION

Our results suggest that sleep deficiency may increase the risk of postmenopausal breast cancer in women. With the exception of duration, linear increases in risk were associated with all individual components of sleep deficiency and with our global sleep index. Due to the high degree of correlation between the individual components of sleep, it is not possible to discern which component(s) of sleep deficiency are driving such risk. Our findings, however, indicate that deficiencies in sleep duration are not likely to be the primary driver of risk.

Most studies to date on this topic have focused on sleep duration. Our results add to this relatively small and somewhat mixed literature.[27, 29–34, 36, 48–52] Overall, the lack of an association between sleep duration and breast cancer risk in our study is consistent with the conclusions of several meta-analyses that found no significant association for either long or short sleep.[15, 16, 18, 19] However, there remains some uncertainty regarding the relationship of long sleep, as suggested by our own results and those from a recent meta-analysis conducted by Lu and colleagues.[17] The Lu meta-analysis used restricted cubic spline modeling to evaluate the shape of the dose-response relationship and reported a pooled relative risk estimate of 1.11 (95% CI: 1.03–1.19) for sleep durations of 10 or more hours.[17] This meta-analysis included results from a prior prospective analysis in the CTS that suggested increased breast cancer risks for very long sleepers based on broadly-defined categories of sleep duration ascertained at the time of cohort entry (hazard ratio = 1.25, 95% CI: 0.93–0.68 for 10+ hours compared to 7–9 hours).[34] In our current analysis, we initially observed a statistically significant elevated OR for long sleep but it was diminished and lost statistical significance in our fully adjusted models. In their meta-analysis, Lu et al. reported that the pooled estimate of risk associated with long sleep duration was more pronounced in cases with ER+ tumors. Consistent with this, although not statistically significant, we observed higher risks associated with long sleep among cases whose tumors were ER+/PR+ than those with ER−/PR− (Supplemental Table 2). Our findings, however, were hampered by the small number of ER−/PR− cases with long durations of sleep (n=15). While overall our findings on sleep duration do not provide evidence that insufficient sleep duration is related to breast cancer risk, the risks associated with long sleep duration may warrant further investigation.

Other than for sleep duration, our results provide evidence for increased breast cancer risks associated with other components of sleep deficiency, including quality, latency and disturbance. Our results add to a small body of inconsistent findings on this topic. They stand in contrast to findings from a handful of breast cancer studies that have examined these other components of sleep deficiency and reported null effects. [29, 32, 33, 36] Although we did not have data available to directly evaluate risks associated with sleep disorders, such as apnea, we did find an increased risk associated with sleep disturbance. As sleep disturbance is considered a hallmark of apnea, our findings are consistent with studies that have reported elevated risks of breast cancer associated with sleep apnea. [23, 24, 26] Our findings are also consistent with the prospective analyses from the NIEHS Sister Study that found some evidence of risk associated with sleep deficiency. Specifically, they reported that relative to women with no difficulty sleeping, those who reported difficulty more than four nights a week were at an approximate 30% increased risk for breast cancer – an effect that was more pronounced among postmenopausal women (HR = 1.51, 95% CI: 1.24–1.85). [36]

Although a number of plausible mechanisms have been suggested, one of the prevailing hypotheses is that cancer risks associated with sleep deficiencies are driven by disruption in circadian rhythms mediated by reductions in melatonin due to light-at-night exposures.[7, 13, 15] In this context, sleep duration has been considered a proxy for light-at-night exposures. Although supported by strong laboratory evidence, the light-at-night hypothesis has not been confirmed in human populations.[53] Circadian disruption, however, is not solely driven by light-at-night exposures and improved measures in epidemiologic studies are needed. As noted in a recent review, [53] measurements of light exposures that incorporate the timing, intensity and spectral qualities of light throughout both the day and night would be highly valuable. Furthermore, integration of actigraphy data with information on light exposures could be used to characterize the synchronization of activity-rest cycles with light-dark exposures, allowing for a more meaningful measure of circadian disruption.

Overall, much remains to be learned about the pathophysiology of sleep and cancer.[20, 55] As noted in a recent review, the degree to which sleep directly impacts breast cancer risk, independent of disruptions in circadian rhythm, is difficult to discern because breast cancer studies typically have not simultaneously considered the impact of both sleep and circadian disruption and the possible interaction of the two.[20] In this review, Samuelsson and colleagues present a nice discussion of the bidirectional relationship between sleep patterns and the circadian system in which each affects the other and together contribute to circadian disruption. While we did not have information on circadian disruption for our study population, we did have information on chronotype. Chronotype (the behavioral manifestation of an individual’s underlying circadian rhythms), is primarily characterized by one’s propensity to sleep at a particular time during the 24-hour cycle (e.g., morning larks and night owls). Research among night shift workers suggests that chronotype may act as a susceptibility factor for circadian disruption.[56–60] Prior analyses in the CTS have shown a relationship between chronotype and breast cancer risk.[46] Building on these observations, we stratified our analyses to explore whether chronotype might modify the risks associated with sleep deficiency in our study. These analyses indicated that risks associated with some measures of sleep deficiency were significantly modified by chronotype (Supplemental Table S1). Interpretation of these findings, however, is difficult as there were no apparent and consistent patterns of risk. To our knowledge, only one other breast cancer study has evaluated the role of sleep deficiency in the context of chronotype.[33] While that study reported no evidence of differential risk by chronotype, there was some suggestion that risks may vary by the other characteristics of circadian rhythm (amplitude and stability) – data we did not collect.

With its large sample size, extensive information on covariates, and ascertainment of several metrics of sleep deficiency coupled with information on sleep stability and chronotype, our study offers a valuable contribution to the limited literature on this topic. There are, however, some limitations of our study worth noting. Sleep characteristics were ascertained by self-report and thus may not be accurate measures of sleep deficiency. Although validation studies have indicated moderate to good agreement between self-reported estimates of sleep duration with objectively measured assessments through polysomnography (PSG) or actigraphy, random error and systematic biases also have been noted. [61–64]

As a case control study reliant on self-reported sleep data, we also cannot dismiss the potential for recall bias due to differential recall between cases and controls. A meta-analyses of sleep duration and breast cancer reported that on average, risk estimates have been approximately 16% higher in prospective cohort studies than in case-control studies.[16] This suggests that if our study was affected by recall bias, it is more likely to have resulted in underestimates than overestimates of risk – at least for sleep duration. The post-diagnostic recall of sleep characteristics among cases in our study introduces the potential for reverse causality, i.e, that the onset of breast cancer, or its treatment, caused changes in sleep rather than sleep causing the cancer. The results of our sensitivity analyses, however, provided little evidence of this. Among cases, we did not observe any differences in sleep characteristics among those who had been more recently diagnosed compared to those with more distant diagnoses. Likewise, exclusion of cases who completed the Q5 survey on sleep characteristics shortly after diagnosis (i.e., with one or two years), did not appreciably change the risk estimates for breast cancer. Furthermore, restriction of our analyses to participants who indicated that their reported sleep had not changed in the last year and was indicative of their sleep for at least 5 years or more did not alter the conclusions of our analyses. Although it is well-documented that sleep changes with aging, such changes predominantly occur earlier in life and tend to stabilize by about age 60 or 65. [65] In our study, sleep stability was slightly greater among those aged 65 or older (*data not shown*) but no statistically significant differences in sleep-associated risks were observed for older (age 65+ years) compared to younger (< 65 years) participants.

In summary, our findings provide evidence that sleep deficiency may increase the risk of postmenopausal breast cancer. While our study importantly captured dimensions of sleep latency and disturbance, our analyses did not capture all measures of sleep deficiency and could not directly assess circadian disruption. Furthermore, our analyses did not include an evaluation of sleep disorders, were limited to night-time sleep, and did not consider the timing of sleep in relation to circadian rhythms. Future epidemiologic studies should consider the use of existing actigraphy tools [66] to objectively measure elements of circadian disruption that capture multiple dimensions of sleep deficiency and would allow for an evaluation of the synchrony of sleep-wake activity patterns with detailed measures of light throughout the 24-hour daily cycle. Additionally, the use of inflammatory, metabolic and immunologic biomarkers to detect upstream effects of sleep deficiency could help elucidate the etiologic underpinnings of breast carcinogenesis, as well as inform the development of potential interventions to improve sleep that ultimately could reduce the risk of this disease.

## Supplementary Material

10552_2020_1349_MOESM1_ESM

## Figures and Tables

**Table 1.** Characteristics of the study population (n=41,505), and distribution by case-control status.

Characteristic^a^	Case control status				All		Chi-square p-value
	Non-case		Case				
	N	%	N	%	N	%	
Full Study Population	38649	100	2856	100	41505	100	
Age (years)							<0.001
40–49	742	2	33	1	775	2	
50–59	6699	17	295	10	6994	17	
60–69	16184	42	1018	36	17202	41	
70–79	10076	26	1026	36	11102	27	
80–89	4948	13	484	17	5432	13	
Race/Ethnicity							0.007
White	33624	87	2535	89	36159	87	
Non-White	5025	13	321	11	5346	13	
Chronotype							0.006
Morning type	14406	37	978	34	15384	37	
More morning than evening type	7776	20	568	20	8344	20	
Neither morning/evening type	5031	13	397	14	5428	13	
More evening than morning type	5687	15	434	15	6121	15	
Evening type	4762	12	406	14	5168	12	
Unknown	987	3	73	3	1060	3	
Smoking status at baseline							<0.001
Never	25939	67	1755	61	27694	67	
Former	11102	29	949	33	12051	29	
Current	1444	4	136	5	1580	4	
Unknown	164	0	16	1	180	0	
Smoking pack-years at baseline							<0.001
≤10	6771	18	521	18	7292	18	
11–20	2252	6	201	7	2453	6	
21–30	1190	3	106	4	1296	3	
≥31	1267	3	160	6	1427	3	
Unknown	1230	3	113	4	1343	3	
Never smokers	25939	67	1755	61	27694	67	
							
Alcohol consumption (g/day) at baseline							<0.001
None	11497	30	793	28	12290	30	
<20	22723	59	1670	58	24393	59	
≥20	3055	8	289	10	3344	8	
Unknown	1374	4	104	4	1478	4	
Age at menarche reported (years) at baseline							0.464
≤11	8951	23	691	24	9642	23	
12–13	21929	57	1579	55	23508	57	
≥14	7331	19	551	19	7882	19	
Unknown/N ever	438	1	35	1	473	1	
Age at first full-term pregnancy(years) reported at baseline							0.351
No full-term pregnancy	8760	23	660	23	9420	23	
≤24	10435	27	736	26	11171	27	
25–29	11811	31	916	32	12727	31	
≥30	7101	18	506	18	7607	18	
Unknown	542	1	38	1	580	1	
Breast feeding history(months) reported at baseline							0.017
Never pregnant	6585	17	499	17	7084	17	
Pregnancy, but no live birth	2134	6	158	6	2292	6	
0 months	5559	14	475	17	6034	15	
>0 and <6 months	6437	17	488	17	6925	17	
6–11	5670	15	387	14	6057	15	
≥12	11585	30	803	28	12388	30	
Unknown	679	2	46	2	725	2	
BMI (kg/m^2^)							0.007
15.0–24.0	18595	48	1293	45	19888	48	
25.0–29.0	11068	29	889	31	11957	29	
30.0–54.8	7133	18	520	18	7653	18	
Unknown	1853	5	154	5	2007	5	
Physical activity (strenuous plus moderate, hours/week)							<0.001
0 to <2.38	12485	32	1060	37	13545	33	
2.38 to <5.88	12927	33	932	33	13859	33	
5.88 to 24.00	13112	34	856	30	13968	34	
Unknown	125	0	8	0	133	0	
Family history of breast cancer reported at Q4							<0.001
No	31139	81	2104	74	33243	80	
Yes	6438	17	667	23	7105	17	
Unknown	1072	3	85	3	1157	3	
Age at menopause (years)							0.166
10–39	2890	7	196	7	3086	7	
40–49	10220	26	806	28	11026	27	
50–54	12459	32	872	31	13331	32	
55–59	5065	13	370	13	5435	13	
60–70	661	2	47	2	708	2	
Unknown	7354	19	565	20	7919	19	
Hormone Therapy Use							<0.001
Never	7551	20	493	17	8044	19	
Ever	26856	69	2120	74	28976	70	
Unknown	4242	11	243	9	4485	11	
Diabetes: Ever Diagnosed							0.843
No	35048	91	2581	90	37629	91	
Yes	3260	8	248	9	3508	8	
Unknown	341	1	27	1	368	1	
Chronic obstructive pulmonary disease (COPD): Ever Diagnosed							<0.001
No	36085	93	2612	91	38697	93	
Yes	1078	3	110	4	1188	3	
Unknown	1486	4	134	5	1620	4	
							
Parkinson’s disease: Ever Diagnosed							0.003
No	37032	96	2698	94	39730	96	
Yes	197	1	18	1	215	1	
Unknown	1420	4	140	5	1560	4	
Depression: Ever Diagnosed							0.001
No	29817	77	2171	76	31988	77	
Yes	7262	19	529	19	7791	19	
Unknown	1570	4	156	5	1726	4	
Current use of depression medication							0.009^c^
No	2994	8	183	6	3177	8	
Yes	3264	8	270	9	3534	9	
Unknown medication use	1004	3	76	3	1080	3	
No/unknown depression	31387	81	2327	81	33714	81	
Chronic fatigue syndrome(CFS): Ever Diagnosed							0.001
No	36322	94	2640	92	38962	94	
Yes	820	2	64	2	884	2	
Unknown	1507	4	152	5	1659	4	
Lupus: Ever Diagnosed							0.001
No	36551	95	2658	93	39209	94	
Yes	313	1	22	1	335	1	
Unknown	1785	5	176	6	1961	5	
Inflammatory bowel disease (IBD) or Crohn’s disease: Ever Diagnosed							0.001
No	35635	92	2606	91	38241	92	
Yes	1518	4	100	4	1618	4	
Unknown	1496	4	150	5	1646	4	
Multiple Sclerosis: Ever Diagnosed							<0.001
No	37056	96	2693	94	39749	96	
Yes	207	1	20	1	227	1	
Unknown	1386	4	143	5	1529	4	
Comorbidities^b^							0.001
None	24372	63	1756	61	26128	63	
1	9862	26	703	25	10565	25	
2	1853	5	151	5	2004	5	
≥ 3	331	1	32	1	363	1	
Unknown	2231	6	214	7	2445	6	
Current Pain Medication Use (# tablets/week)							0.065
None or <1/week	35420	92	2586	91	38006	92	
≥ 1	2083	5	183	6	2266	5	
Unknown	1146	3	87	3	1233	3	
Current NSAID use (# tablets/week)							0.466
None or <1/week	15155	39	1105	39	16260	39	
≥ 1	21267	55	1599	56	22866	55	
Unknown	2227	6	152	5	2379	6	
Marital Status							<0.001
Married	24537	63	1742	61	26279	63	
Divorced/Separated	5641	15	391	14	6032	15	
Widowed	5305	14	504	18	5809	14	
Never married	2402	6	169	6	2571	6	
Unknown	764	2	50	2	814	2	
Household income at Q4							<0.001
< $25,000-$49,999	4231	11	383	13	4614	11	
$50,000-$74,999	7656	20	639	22	8295	20	
$75,000-$99,999	7023	18	580	20	7603	18	
$100,000-$149,999	7169	19	452	16	7621	18	
$150,000-$200,000+	4620	12	309	11	4929	12	
Unknown	7950	21	493	17	8443	20	
Neighborhood SES at baseline							0.259
Lowest quartile	1445	4	95	3	1540	4	
2^nd^ quartile	6011	16	433	15	6444	16	
3^rd^ quartile	12352	32	884	31	13236	32	
Highest quartile	18378	48	1415	50	19793	48	
Unknown	463	1	29	1	492	1	

a Unless otherwise noted, assessment was based on the CTS Q5 survey.

b Comorbidity = (sum(Depression,Diabetes,IBD/Crohn’s,COPD,CFS,Parkinson’s,Lupus,MS))

c Among those with reported depression.

**Table 2.** Distribution of sleep deficiency characteristics for entire study population (n=41,505), and by case-control status.

Characteristic	Case control status				All		Chi-square p-value
	Non-case		Case				
	N	%	N	%	N	%	
Sleep quality							0.158
Very good	11788	30	825	29	12613	30	
Fairly good	21014	54	1562	55	22576	54	
Fairly bad	5175	13	407	14	5582	13	
Very bad	488	1	45	2	533	1	
Unknown	184	0	17	1	201	0	
							
Sleep duration (hours)							0.040
≥ 9 hours	1762	5	162	6	1924	5	
8 hours	9982	26	715	25	10697	26	
7 hours	16120	42	1168	41	17288	42	
5–6 hours	9461	24	702	25	10163	24	
< 5 hours	994	3	75	3	1069	3	
Unknown	330	1	34	1	364	1	
							
Sleep latency							<0.001
< 15 minutes	18151	47	1188	42	19339	47	
16–30 minutes	13876	36	1102	39	14978	36	
31–60 minutes	4895	13	416	15	5311	13	
> 60 minutes	1540	4	138	5	1678	4	
Unknown	187	0	12	0	199	0	
							
Sleep disturbance							0.003
Not during past month	8600	22	619	22	9219	22	
< 1 time/week in past month	12579	33	868	30	13447	32	
1–2 times/week in past month	9575	25	701	25	10276	25	
≥ 3 times in past month	7752	20	658	23	8410	20	
Unknown	143	0	10	0	153	0	
Sleep medication							<0.001
Not during past month	26655	69	1841	64	28496	69	
< 1 time/week in past month	4201	11	347	12	4548	11	
1–2 times/week in past month	2257	6	210	7	2467	6	
≥ 3 times in past month	5200	13	427	15	5627	14	
Unknown	336	1	31	1	367	1	
							
Global Sleep Index (GSI)							<0.001
Lowest quartile (better sleep)	10853	28	738	26	11591	28	
2nd quartile	9550	25	642	22	10192	25	
3rd quartile	7540	20	587	21	8127	20	
Highest quartile (worse sleep)	9655	25	792	28	10447	25	
Unknown	1051	3	97	3	1148	3	
							
Sleep stability past year							0.060
No	2790	7	216	8	3006	7	
Yes	34061	88	2481	87	36542	88	
Unknown	1798	5	159	6	1957	5	
							
Sleep stability past 2–5 years							0.161
No	8256	21	601	21	8857	21	
Yes	27929	72	2047	72	29976	72	
Unknown	2464	6	208	7	2672	6	
							
Sleep stability past 6–10 year							0.254
No	16408	42	1206	42	17614	42	
Yes	19372	50	1414	50	20786	50	
Unknown	2869	7	236	8	3105	7	
							
Sleep stability past 11 or more years							0.022
No	19296	50	1449	51	20745	50	
Yes	16758	43	1183	41	17941	43	
Unknown	2595	7	224	8	2819	7	

**Table 3.** Sleep DeficiencyVariables: Correlation Matrix

	Spearman Rank Correlation Coefficient^a^				
	Sleep Latency	Sleep Disturbance	Sleep Duration	Sleep Medication	Global Sleep Index
Sleep Quality	0.44	0.66	0.46	0.28	0.75
Sleep Latency		0.45	0.24	0.27	0.63
Sleep Disturbance			0.39	0.29	0.79
Sleep Duration				0.10	0.56
Sleep Medication					0.55

a all are statistically significant at p < 0.001

**Table 4.** Risk of breast cancer associated with sleep deficiency, estimated by logistic regression analyses among full study population (n=41,505).

Sleep metric		Adjusted for age and race		Fully adjusted^a^
	N cases^b^ (n=2,856)	OR (95% CI)		OR (95% CI)
Sleep quality				
Very good	825	1.00		1.00
Fairly good	1562	1.11 (1.02, 1.21)		1.10 (1.01, 1.20)
Fairly bad	407	1.20 (1.06, 1.36)		1.18 (1.04, 1.33)
Very bad	45	1.35 (0.99, 1.85)		1.32 (0.96, 1.82)
*p-value for trend*		*0.001*		*0.002*
				
Sleep latency				
< 15 minutes	1188	1.00		1.00
16–30 minutes	1102	1.19 (1.10, 1.30)		1.19 (1.09, 1.30)
31–60 minutes	416	1.31 (1.17, 1.48)		1.30 (1.15, 1.46)
> 60 minutes	138	1.36 (1.13, 1.63)		1.33 (1.11, 1.61)
*p-value for trend*		*<0.001*		*<0.001*
				
Sleep disturbance				
Not during past month	619	1.00		1.00
< 1 time/week in past month	868	1.00 (0.90, 1.11)		1.00 (0.90, 1.12)
1–2 times/week in past month	701	1.07 (0.96, 1.20)		1.08 (0.96, 1.21)
≥ 3 times in past month	658	1.25 (1.11, 1.40)		1.25 (1.11, 1.40)
*p-value for trend*		*<0.001*		*<0.001*
				
Sleep duration				
≥ 9 hours	162	1.22 (1.02, 1.46)		1.17 (0.98, 1.41)
8 hours	715	1.00		1.00
7 hours	1168	1.04 (0.94, 1.14)		1.05 (0.95, 1.16)
5–6 hours	702	1.06 (0.95, 1.19)		1.07 (0.96, 1.20)
< 5 hours	75	1.06 (0.83, 1.36)		1.05 (0.82, 1.35)
*p-value for trend*		*0.997*		*0.775*
Sleep medication				
Not during past month	1841	1.00		1.00
<1 time/week	347	1.23 (1.09, 1.38)		1.24 (1.10, 1.40)
1–2 time/week	210	1.36 (1.17, 1.58)		1.36 (1.17, 1.58)
3+ times/week	427	1.17 (1.05, 1.30)		1.15 (1.03, 1.29)
*p-value for trend*		*<0.001*		*<0.001*
				
Global Sleep Index (GSI)				
Lowest quartile (better sleep)	738	1.00		1.00
2nd quartile	642	1.03 (0.92, 1.15)		1.02 (0.92, 1.14)
3rd quartile	587	1.18 (1.06, 1.32)		1.18 (1.05, 1.32)
Highest quartile (worse sleep)	792	1.25 (1.13, 1.39)		1.24 (1.12, 1.38)
*p-value for trend*		*<0.001*		*<0.001*

a Adjusted for age at Q5, race (white/non-white), total pack-years of smoking, age at first full-term pregnancy, BMI at Q5, physical activity at Q5, family history of breast cancer through Q4, age at menopause (calculated at Q5), medication use for depression at Q5, NSAID use at Q5, and marital status at Q5.

b Numbers do not sum to total due to missing/unknown values.

**Table 5.** Sensitivity analysis: estimated risk of breast cancer associated with global sleep index (GSI), estimated by multivariable logistic regression, applying various exclusions.

Study Population	Adjusted Odds Ratios (95% CI)^a^ Global Sleep Index (GSI)			
	1^st^ Quartile (better sleep)	2^nd^ Quartile	3^rd^ Quartile	4^th^ Quartile (worse sleep)
Full Study Population (n=41,505)	1.00	1.02 (0.92, 1.14)	1.18 (1.05, 1.32)	1.24 (1.12, 1.38)
Excluding 363 cases diagnosed within 2 years of Q5 fill date	1.00	0.98 (0.88, 1.11)	1.13 (1.00, 1.27)	1.20 (1.07, 1.34)
Excluding 178 cases diagnosed within 1 year of Q5 fill date	1.00	1.00 (0.89, 1.12)	1.15 (1.02, 1.29)	1.20 (1.08, 1.34)
Restricted to those with no recent changes in sleep^b^ (n=36,542)	1.00	1.02 (0.91, 1.14)	1.17 (1.03,1.31)	1.16 (1.04,1.30)
Restricted to those who report long-term sleep stability^c^ (=28,714)	1.00	1.03 (0.91, 1.17)	1.23 (1.08,1.41)	1.16 (1.02,1.32)

a Adjusted for age at Q5, race (white/non-white), total pack-years of smoking, age at first full-term pregnancy, BMI at Q5, physical activity at Q5, family history of breast cancer through Q4, age at menopause (calculated at Q5), medication use for depression at Q5, NSAID use at Q5, and marital status at Q5.

b Reported sleep habits were typical of the prior year.

c Reported sleep habits were typical of at least the prior 5 years.
