# Supplementary material for: Sleep Deficiency and Breast Cancer Risk Among Postmenopausal Women in the California Teachers Study (CTS)
Source: Cancer Causes Control. Author manuscript; Available in PMC 2021 Dec 1. (PMC8519507; doi:10.1007/s10552-020-01349-2)
Supplement: 10552_2020_1349_MOESM1_ESM [file NIHMS1632800-supplement-10552_2020_1349_MOESM1_ESM.docx]

**SUPPLEMENTAL TABLES**

**TITLE:** Sleep Deficiency and Breast Cancer Risk Among Postmenopausal Women in the California Teachers Study (CTS)

**AUTHORS:** S Hurley^1,*^, D Goldberg^1^, J Von Behren^1^, J Clague DeHart^2^, S Wang^3^, P Reynolds^1^

^1^Department of Epidemiology and Biostatistics, University of California San Francisco, Berkeley, CA, USA

^2^ School of Community and Global Health, Claremont Graduate University, Claremont, CA, USA

^3^ Division of Health Analytics, Department of Computational and Quantitative Medicine, Beckman Research Institute, City of Hope Comprehensive Cancer Center, Duarte, CA, USA

^*^ Corresponding author: susan.hurley@oehha.ca.gov; 925-608-5189. ORCID: 0000-0002-5582-1741

**Table S1. Risk of breast cancer associated with sleep deficiency among postmenopausal CTS participants, estimated by multivariable logistic regression, stratified by chronotype^.* ±^**

| Sleep metric | Morning type | |  | Evening type | |  | Neither type | | Interaction  p-value |
| --- | --- | --- | --- | --- | --- | --- | --- | --- | --- |
|  | N cases | OR* (95% CI) |  | N cases | OR* (95% CI) |  | N cases | OR* (95% CI) |  |
| Sleep quality |  |  |  |  |  |  |  |  | 0.710 |
| Very good | 459 | 1.00 |  | 232 | 1.00 |  | 117 | 1.00 |  |
| Fairly good | 861 | 1.09 (0.97, 1.23) |  | 450 | 1.08 (0.91, 1.27) |  | 207 | 1.17 (0.92, 1.48) |  |
| Fairly/Very bad | 221 | 1.16 (0.98, 1.37) |  | 151 | 1.15 (0.93, 1.43) |  | 69 | 1.43 (1.04, 1.97) |  |
| Sleep latency |  |  |  |  |  |  |  |  | 0.521 |
| < 15 minutes | 696 | 1.00 |  | 297 | 1.00 |  | 167 | 1.00 |  |
| 16-30 minutes | 601 | 1.21 (1.08, 1.36) |  | 339 | 1.26 (1.07, 1.48) |  | 141 | 1.04 (0.83, 1.32) |  |
| 31-60 minutes | 195 | 1.30 (1.10, 1.53) |  | 140 | 1.19 (0.97, 1.48) |  | 63 | 1.38 (1.01, 1.89) |  |
| >60 minutes | 48 | 1.29 (0.95, 1.76) |  | 60 | 1.27 (0.95, 1.71) |  | 24 | 1.74 (1.10, 2.77) |  |
| Sleep disturbance |  |  |  |  |  |  |  |  | 0.032 |
| Not during past month | 318 | 1.00 |  | 192 | 1.00 |  | 93 | 1.00 |  |
| < 1 time/wk in past month | 495 | 1.05 (0.91, 1.22) |  | 252 | 1.00 (0.82, 1.22) |  | 99 | 0.81 (0.60, 1.09) |  |
| 1-2 times/wk in past month | 379 | 1.05 (0.90, 1.22) |  | 187 | 1.00 (0.81, 1.24) |  | 117 | 1.39 (1.04, 1.87) |  |
| 3+ times/wk in past month | 351 | 1.26 (1.08, 1.48) |  | 205 | 1.25 (1.01, 1.54) |  | 87 | 1.27 (0.93, 1.74) |  |
| Sleep duration |  |  |  |  |  |  |  |  | 0.166 |
| 9+ hours | 76 | 1.18 (0.91, 1.54) |  | 53 | 1.12 (0.81, 1.55) |  | 27 | 1.27 (0.81, 1.98) |  |
| 8 hours | 400 | 1.00 |  | 179 | 1.00 |  | 122 | 1.00 |  |
| 7 hours | 637 | 0.99 (0.87, 1.13) |  | 359 | 1.27 (1.05, 1.53) |  | 148 | 0.90 (0.70, 1.16) |  |
| 5-6 hours | 384 | 1.04 (0.89, 1.20) |  | 205 | 1.17 (0.95, 1.45) |  | 89 | 0.96 (0.72, 1.29) |  |
| <5 hours | 32 | 0.87 (0.60, 1.27) |  | 31 | 1.50 (1.00, 2.26) |  | 8 | 0.75 (0.36, 1.58) |  |
| Sleep medication |  |  |  |  |  |  |  |  | <0.001 |
| Not during past month | 1000 | 1.00 |  | 538 | 1.00 |  | 256 | 1.00 |  |
| <1 time/week | 201 | 1.30 (1.11, 1.53) |  | 113 | 1.40 (1.13, 1.74) |  | 27 | 0.73 (0.49, 1.11) |  |
| 1-2 time/week | 95 | 1.17 (0.93, 1.45) |  | 70 | 1.44 (1.10, 1.87) |  | 38 | 2.11 (1.45, 3.07) |  |
| 3+ times/week | 236 | 1.28 (1.10, 1.49) |  | 109 | 0.89 (0.71, 1.10) |  | 72 | 1.49 (1.12, 1.98) |  |
| Global Sleep Index (GSI) |  |  |  |  |  |  |  |  | 0.669 |
| Lowest quartile (better sleep) | 405 | 1.00 |  | 205 | 1.00 |  | 113 | 1.00 |  |
| 2nd quartile | 367 | 1.07 (0.92, 1.24) |  | 178 | 0.97 (0.79, 1.20) |  | 78 | 0.88 (0.65, 1.19) |  |
| 3rd quartile | 325 | 1.17 (1.00, 1.36) |  | 174 | 1.22 (0.99, 1.51) |  | 78 | 1.19 (0.88, 1.61) |  |
| Highest quartile (Worse sleep) | 407 | 1.25 (1.08, 1.44) |  | 246 | 1.18 (0.97, 1.44) |  | 116 | 1.41 (1.07, 1.86) |  |

*Adjusted for age at Q5, race (white/non-white), total pack-years of smoking, age at first full-term pregnancy, BMI at Q5, physical activity at Q5, family history of breast cancer through Q4, age at menopause (calculated at Q5), medication use for depression at Q5, NSAID use at Q5, and marital status at Q5;

^±^ excludes n=1,062 with unknown chronotype

**Table S2. Risk of hormonally responsive (ER+ or PR+) and non-hormonally responsive (ER- and PR-) breast cancer associated with sleep deficiency among postmenopausal CTS participants, estimated by multivariable polytomous logistic regression.^* ±^**

| Sleep metric | Cases: ER or PR positive | |  | Cases: ER and PR negative | |
| --- | --- | --- | --- | --- | --- |
|  | N cases†  (n=2,262) | Multivariate*  OR (95% CI) |  | N cases  (n=343) | Multivariate*  OR (95% CI) |
| Sleep quality |  |  |  |  |  |
| Very good | 654 | 1.00 |  | 85 | 1.00 |
| Fairly good | 1236 | 1.10 (1.00, 1.22) |  | 200 | 1.32 (1.02, 1.71) |
| Fairly/Very bad | 357 | 1.19 (1.04, 1.36) |  | 57 | 1.38 (0.99, 1.95) |
| Sleep latency |  |  |  |  |  |
| < 15 minutes | 961 | 1.00 |  | 118 | 1.00 |
| 16-30 minutes | 867 | 1.16 (1.05, 1.27) |  | 148 | 1.62 (1.27, 2.07) |
| 31-60 minutes | 318 | 1.22 (1.07, 1.39) |  | 60 | 1.86 (1.36, 2.55) |
| >60 minutes | 108 | 1.29 (1.04, 1.59) |  | 14 | 1.34 (0.76, 2.35) |
| Sleep disturbance |  |  |  |  |  |
| Not during past month | 507 | 1.00 |  | 60 | 1.00 |
| <1 time/week | 671 | 0.95 (0.84, 1.07) |  | 103 | 1.20 (0.87, 1.66) |
| 1-2 time/week | 551 | 1.04 (0.92, 1.18) |  | 100 | 1.53 (1.11, 2.12) |
| 3+ times/week | 524 | 1.22 (1.07, 1.38) |  | 80 | 1.50 (1.07, 2.11) |
| Sleep duration |  |  |  |  |  |
| 9+ hours | 133 | 1.21 (0.99, 1.47) |  | 15 | 0.96 (0.55, 1.68) |
| 8 hours | 564 | 1.00 |  | 82 | 1.00 |
| 7 hours | 925 | 1.06 (0.95, 1.18) |  | 152 | 1.17 (0.89, 1.53) |
| 5-6 hours | 562 | 1.09 (0.96, 1.23) |  | 79 | 1.03 (0.76, 1.42) |
| <5 hours | 54 | 0.96 (0.72, 1.28) |  | 8 | 0.96 (0.46, 1.99) |
| Sleep medication |  |  |  |  |  |
| Not during past month | 1453 | 1.00 |  | 214 | 1.00 |
| <1 time/week | 264 | 1.20 (1.05, 1.38) |  | 53 | 1.58 (1.17, 2.15) |
| 1-2 time/week | 163 | 1.34 (1.13, 1.59) |  | 30 | 1.63 (1.10, 2.40) |
| 3+ times/week | 355 | 1.21 (1.07, 1.37) |  | 44 | 1.01 (0.73, 1.41) |
| Global Sleep (GSI) |  |  |  |  |  |
| Lowest quartile (better sleep) | 584 | 1.00 |  | 75 | 1.00 |
| 2nd quartile | 508 | 1.03 (0.91, 1.16) |  | 81 | 1.24 (0.90, 1.70) |
| 3rd quartile | 478 | 1.21 (1.07, 1.38) |  | 64 | 1.23 (0.88, 1.72) |
| Highest quartile (Worse sleep) | 615 | 1.22 (1.08, 1.37) |  | 110 | 1.64 (1.22, 2.22) |

*Adjusted for age at Q5, race (white/non-white), total pack-years of smoking, age at first full-term pregnancy, BMI at Q5, physical activity at Q5, family history of breast cancer through Q4, age at menopause (calculated at Q5), medication use for depression at Q5, NSAID use at Q5, and marital status at Q5.

^±^ Excludes n=251 cases with unknown ER or PR tumor status.

†Numbers do not sum to total due to missing/unknown values of sleep factor.
